# High-Order Modes Micro-Knot Excited by a Long-Period Fiber Grating

**DOI:** 10.3390/s17112490

**Published:** 2017-10-30

**Authors:** Shir Shahal, Hamootal Duadi, Moti Fridman

**Affiliations:** Faculty of Engineering and the Institute of Nanotechnology and Advanced Materials, Bar-Ilan University, Ramat Gan 5290002, Israel; shir.shahal@biu.ac.il (S.S.); hamootal.duadi@biu.ac.il (H.D.)

**Keywords:** fiber micro-knot, long-period fiber grating, high-order fiber modes

## Abstract

We suggest a fiber micro-knot fabricated on a long-period fiber grating. The long-period fiber grating excites high-order modes into the micro-knot and transfers the output back to the Gaussian mode. We show theoretically and experimentally that these micro-knots have an improved Q-factor, higher stability, and have an increased evanescence wave coupling to the environment than single mode fiber micro-knots. These high-order fiber micro-knots can be beneficial for various fiber detectors and optical data processing systems.

Tapered fiber micro-knots serve as ring resonators thanks to evanescence wave coupling between touching tapered fibers [1]. These fiber micro-knots are easily fabricated and proved to be sensitive to external conditions such as temperature, motion, and humidity, which made them idle for a variety of optical detectors [2,3,4,5,6,7,8,9]. However, when designing a micro-knot, there is a trade-off between thin fibers that have stronger evanescence wave coupling and thick fibers that are more robust and stable [10]. Thin fibers with higher evanescence wave coupling have both higher sensitivity to the environment and higher Q-factor, but they tend to break. We suggest to excite high-order modes in micro-knots fabricated on thick and robust fibers, since high-order modes have stronger evanescence wave coupling [11,12]. We will excite the high-order modes in the micro-knot by a long-period fiber grating written on a tapered fiber [13]. We present calculated and measured results of such fiber micro-knots and their improved parameters. We believe that such micro-knots can lead to cheaper and more accurate fiber-based detectors.

In tapered fibers, the light is guided by the cladding, resulting in evanescence wave coupled to the environment. The calculated evanescence wave coupling as a function of the fiber diameter is shown in Figure 1b. As is evident, the coupling strength grows exponentially for thinner fibers and reaches a value of more than 20% for 2.5 μm-thick fibers; however, these fibers are also fragile and tend to break. We overcome this drawback by resorting to thick and robust fibers and exciting high-order modes for increasing efficiency. Thick tapered fibers support high-order modes which have stronger evanescence wave coupling than the low-order modes in thinner fibers. Calculated evanescence wave coupling as a function of the mode for fibers with different diameters is shown in Figure 1a. Specifically, we present the evanescence wave coupling as a function of the propagation vector for the first few modes for 4, 6, 8, and 10 μm-thick fibers. The coupling strength for high-order modes can reach 30% regardless of the fiber thickness proving that thick fibers excited with high-order modes can have strong evanescence wave coupling.

Several methods for exciting high-order modes in fibers were presented, such as: off-center splicing, external mechanical pressure, and long-period fiber gratings (LPFGs) [14,15,16]. LPFGs are based on a periodic structure written on a fiber with a much longer periodicity than the wavelength [17,18]. This long periodicity leads to a small momentum kick which is idle for exciting different modes in fibers due to the small momentum difference between the modes [19,20].

We suggest the fabrication of a micro-knot from a LPFG, as presented in Figure 2a. In such micro-knots, the first part of the LPFG excites the input Gaussian mode to a high-order mode. This high-order mode resonates in the micro-knot with improved coupling strength. The output from the micro-knot is excited back to the Gaussian mode by the second part of the LPFG. Exciting high-order modes in a micro-knot leads to a strong evanescence wave coupling with the environment and improved Q-factor, which is important for sensitive detectors. We are assuming that the periodicity of the LPFG is longer than the length of the micro-knot. When this assumption is not valid, a coupling between different modes occurs inside the micro-knot, which is also discussed in this letter.

We compared the calculated spectral responses of a micro-knot fabricated on a 6 μm-wide fiber excited with two different modes: the Gaussian, $HE_{11}$ mode and the higher $TE_{01}$ mode, presented in Figure 2b. The results show that the amplitude of the spectral fluctuations of the high-order mode are 2.4 times larger than the Gaussian mode, resulting from the stronger evanescence wave coupling. We also note that the free spectral range for high-order mode is shorter due to its smaller propagation vector [12]. A microscope image of a micro-knot fabricated on a LPFG is shown in Figure 2c.

We analyzed the LPFG spectral response of a 4-mm LPFG with periodicity of 180 μm as a function of the LPFG length following Erdogan derivation [21]. Specifically, we numerically calculated the transmission spectrum by resorting to:

(1) $$t_{=}=\cos^{2}\left(\sqrt[{}]{\kappa^{2}+{\hat{\sigma }}^{2}}z\right)+\frac{{\hat{\sigma }}^{2}}{\kappa^{2}+{\hat{\sigma }}^{2}}\sin^{2}\left(\sqrt[{}]{\kappa^{2}+{\hat{\sigma }}^{2}}z\right),$$

where $\hat{\sigma }$ is the self-coupling coefficient, and κ is the cross-coupling between the modes in the fiber. The calculated results are presented in Figure 3. The spectral response shows that after 2 mm and between 1559 nm and 1565 nm the light is excited to a high-order mode. Then, after another 2 mm of LPFG, the light is excited back to the Gaussian mode. This area in Figure 3 is emphasized and marked with the white arrows. 

We fabricated two LPFGs from 6 μm-thick fibers—one with a periodicity of 350 μm and the other with a periodicity of 180 μm. These parameters result in spectral oscillations of 8 nm for the first LPFG and 13 nm for the second one [21]. The fabrication was done by tapering the fiber and increasing the power extensively, which causes the fiber to oscillate during the tapering process. These oscillations resulted in a periodic structure written on the tapered fiber [13]. The spectral responses of the LPFGs are presented in Figure 4 as the dashed blue curves, where the spectral response of the first LPFG is shown in Figure 4a and the second one in Figure 4b. We attribute the lower-amplitude spectral oscillations to additional unintended weaker LPFGs with longer periodicity written on the tapered fibers. Next, we knotted micro-knots of both LPFGs, where in the first LPFG we knotted a 1.3 mm-diameter micro-knot and on the second one we knotted a 950 μm-diameter micro-knot leading to a free-spectral range of 0.4 nm and 0.53 nm, respectively. We measured the spectral responses of the LPFGs with the micro-knots and show them in Figure 4 as the solid red curves. The measured results agree with the calculated ones.

The measured spectra of the LPFGs with the micro-knots show both the spectral response of the LPFGs and the spectral response of the micro-knots. At the resonances of the LPFG, the light in the fiber is excited to high-order modes. It is evident that the amplitudes of the spectral oscillations are between three-to-four times larger at a LPFG resonance than the amplitude out of a resonance, as emphasized by the ellipses in Figure 4a. Similar results are observed in the second LPFG, as seen in Figure 4b, where the amplitude of the spectral oscillations is at least twice larger during the LPFG resonance than out of the resonance. The dashed ellipses in Figure 4a highlights part of the spectrum where large amplitude is expected but small amplitude is measured. This can result from interferences with the unintended weak LPFGs which were also written on the tapered fiber.

We analyzed the Q-factor of every resonance in the first micro-knot and plot the Q factor as a function of wavelength in Figure 5 together with the micro-knot and the LPFG spectral responses. The results show that the peaks in the Q-factor agree with the resonance of the LPFG and that the Q-factor of the micro-knot is between four-to-eight times higher for wavelengths at the LPFG resonances. This increase in the Q-factor indicates that micro-knots excited with multi-modes will have an increased coupling to the environment, even for thick fibers.

So far, we neglected the periodicity of the LPFG inside the micro-knot. This is justified only if the micro-knot cavity is shorter than the periodicity of the LPFG. However, in our experiments the cavity length is longer than the periodicity of the LPFG. This creates coupling between different modes in the cavity. We analyzed that by considering the coupling between different modes, $t_{\times }$, according to [13,21]:

(2) $$t_{\times }\left(l\right)=\frac{{\hat{\sigma }}^{2}}{\kappa^{2}+{\hat{\sigma }}^{2}}\sin^{2}\left(\sqrt[{}]{\kappa^{2}+{\hat{\sigma }}^{2}}l\right),$$

where *l* is the length of the cavity. Next, we considered the micro-knot as two cavities, each with a different propagation vector according to the specific mode. The two cavities are coupled by the LPFG, so the effective reflectivity of the coupled cavities [22,23,24] is:

(3) $$E_{out}=\frac{1-\kappa_{1}\left(1-t_{\times }\left(l\right)\right)}{1-\kappa_{1}\left(1-t_{\times }\left(l\right)\right)e^{i\beta_{1}l}}\frac{1-\kappa_{2}\left(1-t_{\times }\left(l\right)\right)}{1-\kappa_{2}\left(1-t_{\times }\left(l\right)\right)e^{i\beta_{2}l}}$$

where $\kappa_{1,2}$ are the coupling strengths between touching fibers and $\beta_{1,2}$ are the propagation parameters for the two modes, respectively. Analyzing Equation (3) reveals that when considering the LPFG in the micro-knot, the main differences in the spectral response are additional spectral oscillations. These additional spectral oscillations can explain the difference between the ordered spectral oscillations at the left ellipse compared to the chaotic spectral oscillations at the right ellipse in Figure 4.

To conclude, we showed that when fabricating micro-knot from a LPFG, the micro-knot frequency response is stronger at the LPFG resonance due to the excitation of high-order modes in the micro-knot. We showed that the Q-factor of the micro-knot resonator increased by a factor of four-to-eight while still utilizing thick and robust fiber. Therefore, high-order modes in micro-knots fabricated on thick LPFGs can improve future fiber detectors.

## Figures and Tables

**Figure 1:**
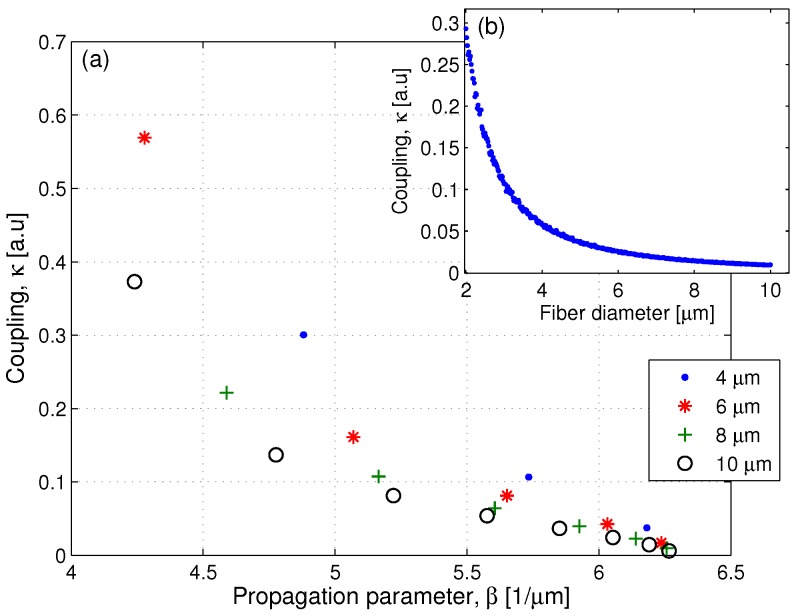
Calculated evanescence wave coupling strength. (**a**) Evanescence wave coupling strength as a function of the propagation vector for fibers with different diameters showing an increased coupling strength for high-order modes. (**b**) Evanescence wave coupling strength of the Gaussian mode as a function of the fiber thickness showing an exponential increased coupling strength for thinner fibers.

**Figure 2:**
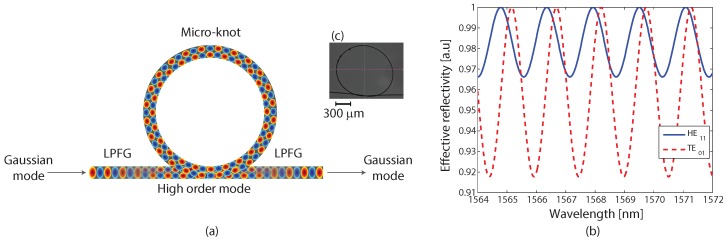
(**a**) Schematics of a micro-knot fabricated from a long-period fiber grating (LPFG). The LPFG excites the input Gaussian mode to a high-order mode that oscillates in the micro-knot and excites back to the Gaussian mode at the output. (**b**) Calculated spectral response of a micro-knot excited by two different modes: Gaussian $HE_{11}$ mode denoted by the solid curve and the higher $TE_{01}$ mode denoted by the dashed curve. (**c**) A microscope image of a fiber micro-knot fabricated from a LPFG.

**Figure 3:**
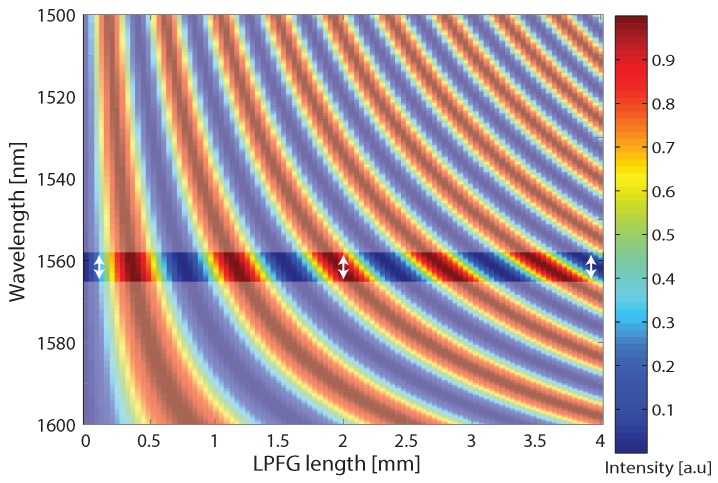
Spectral response of a LPFG with periodicity of 180 μm as a function of the LPFG length. The results show that between 1559 nm and 1565 nm (denoted with white arrows), the light is excited to a high-order mode after 2 mm and then excited back to the Gaussian mode after another 2 mm.

**Figure 4:**
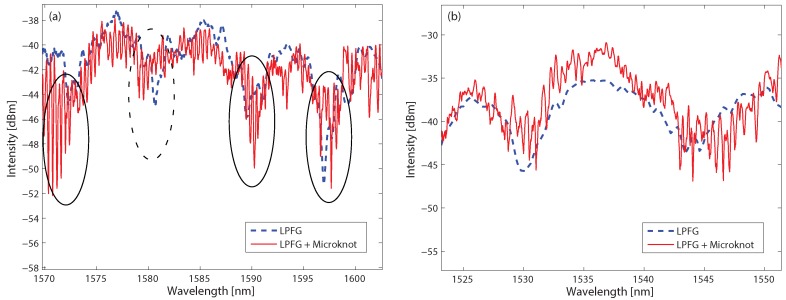
Transmission spectra of LPFGs before knotting the micro-knot denoted by the dashed blue curve and with the micro-knot denoted by the solid red curve. (**a**) LPFG of 5 mm long and a periodicity of 350 μm, micro-knot diameter of 1.3 mm; (**b**) LPFG of 6.5 mm long and a periodicity of 180 μm, micro-knot diameter of 950 μm.

**Figure 5:**
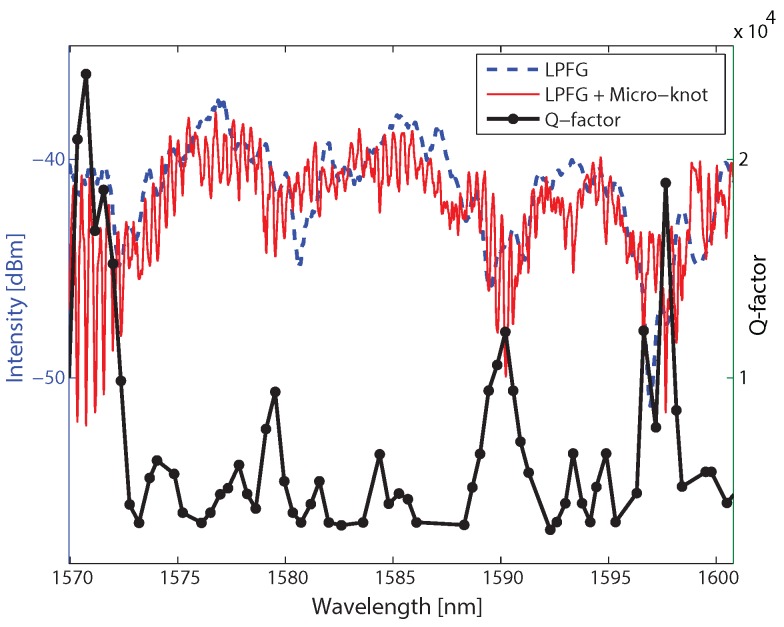
Measured Q-factor of the micro-knot as a function of the wavelength. The increase in the Q-factor of the micro-knot at the LPFG resonances indicates that high-order modes have stronger evanescence wave coupling.
